# In Vitro Synergism of Azithromycin Combination with Antibiotics against OXA-48-Producing *Klebsiella pneumoniae* Clinical Isolates

**DOI:** 10.3390/antibiotics10121551

**Published:** 2021-12-17

**Authors:** Uthaibhorn Singkham-in, Netchanok Muhummudaree, Tanittha Chatsuwan

**Affiliations:** 1 Department of Microbiology, Faculty of Medicine, Chulalongkorn University, Bangkok 10330, Thailand; singkhamin.u@gmail.com; 2 Interdisciplinary Program of Medical Microbiology, Graduate School, Chulalongkorn University, Bangkok 10330, Thailand; netchanok.mu@hotmail.com; 3 Antimicrobial Resistance and Stewardship Research Unit, Faculty of Medicine, Chulalongkorn University, Bangkok 10330, Thailand

**Keywords:** *Klebsiella pneumoniae*, carbapenem resistance, OXA-48, OmpK35, OmpK36, carbapenem, colistin, azithromycin

## Abstract

Carbapenem-resistant *Klebsiella pneumoniae* has globally emerged as an urgent threat leading to the limitation for treatment. *K. pneumoniae* carrying *bla*_OXA-48_, which plays a broad magnitude of carbapenem susceptibility, is widely concerned. This study aimed to characterize related carbapenem resistance mechanisms and forage for new antibiotic combinations to combat *bla*_OXA-48_-carrying *K. pneumoniae*. Among nine isolates, there were two major clones and a singleton identified by ERIC-PCR. Most isolates were resistant to ertapenem (MIC range: 2–>256 mg/L), but two isolates were susceptible to imipenem and meropenem (MIC range: 0.5–1 mg/L). All *bla*_OXA-48_-carrying plasmids conferred carbapenem resistance in *Escherichia coli* transformants. Two ertapenem-susceptible isolates carried both outer membrane proteins (OMPs), OmpK35 and OmpK36. Lack of at least an OMP was present in imipenem-resistant isolates. We evaluated the in vitro activity of an overlooked antibiotic, azithromycin, in combination with other antibiotics. Remarkably, azithromycin exhibited synergism with colistin and fosfomycin by 88.89% and 77.78%, respectively. Bacterial regrowth occurred after exposure to colistin or azithromycin alone. Interestingly, most isolates were killed, reaching synergism by this combination. In conclusion, the combination of azithromycin and colistin may be an alternative strategy in dealing with *bla*_OXA-48_-carrying *K. pneumoniae* infection during a recent shortage of newly effective antibiotic development.

## 1. Introduction

Carbapenem resistance in Enterobacteriaceae is currently a global concern [1]. Among Enterobacteriaceae, *Klebsiella pneumoniae* plays a major role in resistance to carbapenems [2,3]. Most *K. pneumoniae* isolates are resistant to carbapenem by carbapenemase production [2,4]. Apart from metallo-carbapenemases (such as NDM and IMP) and an Ambler class A carbapenemase (KPC) that strongly hydrolyze carbapenemases, OXA-48 produces a weak carbapenemase activity yet is responsible for a broad range of carbapenem susceptibility [4]. Although *bla*_OXA-48_-carrying *K. pneumoniae* isolates are endemic in Turkey, India, some countries in Europe, Africa, and the Middle East, it has been reported to be widespread worldwide [5,6,7,8]. In our previous study, among carbapenemase-producing *K. pneumoniae*, 13.7% were OXA-48 producers that displayed susceptible and resistant phenotypes [9]. In addition to carbapenemase production, the loss of outer membrane proteins (OMPs) (OmpK35 and OmpK36) which act as the carbapenem entry portal, also plays a role in carbapenem susceptibility [10]. Lack of OmpK35 or OmpK36, together with the production of KPC, extended-spectrum β-lactamases (ESBLs), or AmpC has been reported to confer a higher level of carbapenem resistance [11,12,13]. We, therefore, characterized carbapenem susceptibility and investigated carbapenem-related mechanisms in *bla*_OXA-48_-carrying *K. pneumoniae* isolates.

There are limited treatment options for carbapenemase-producing *K. pneumoniae* infections [14]. Apart from colistin, the last resource of choice, the newly effective agents are urgently needed [3]. Unfortunately, with a shortage of novel antibiotics introduced to the discovery pipeline, the antibiotic combination is inevitably an alternative treatment. Azithromycin, a macrolide antibiotic, inhibits bacterial protein production and is a strong potential agent that can be used to treat certain bacteria such as *Neisseria* species, *Salmonella* species, and *Shigella* species [15]. However, azithromycin is also considered effective in combination with antimicrobial peptides or colistin against other Gram-negative bacteria [16]. We aimed to investigate the in vitro activity of azithromycin in combination with other antibiotics against *bla*_OXA-48_-carrying *K. pneumoniae* clinical isolates. This study could provoke further consideration of using azithromycin combination as a new potential strategy to combat carbapenem-resistant *K. pneumoniae* clinical isolates during the antibiotic era that novel agents and policy are needed to combat antibiotic resistance [17].

## 2. Results

### 2.1. Antibiotic Susceptibility of OXA-48-Producing K. pneumoniae Isolates

Among nine *bla*_OXA-48_-carrying *K. pneumoniae* isolates, seven isolates were resistant to all tested carbapenems (imipenem, meropenem, and ertapenem), whereas KP203 and KP221 isolates were only resistant to ertapenem (Table 1). All isolates appeared susceptible to colistin but resistant to ciprofloxacin and ceftriaxone. Amikacin-resistant strains were KP197 and KP1184. Fosfomycin and azithromycin showed an efficient effect on only three and two isolates, respectively. These results indicate that all isolates were multidrug-resistant (MDR) strains; they were resistant to at least three antibiotic classes, including carbapenems, extended-spectrum cephalosporins, and fluoroquinolones (Table 1). These phenotypes limit the therapeutic option for *bla*_OXA-48_-carrying *K. pneumoniae*.

### 2.2. Two Major Clonalities of OXA-48-Producing K. pneumoniae

**Figure 1:**
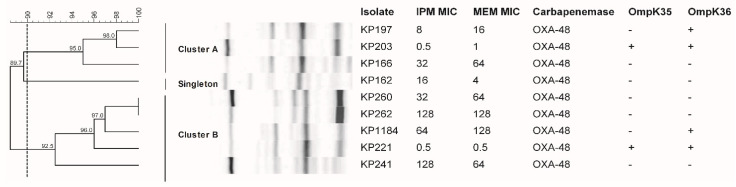
Genetic similarity of OXA-48-producing *K. pneumoniae* isolates. Dendrogram of genetic similarity based on ERIC-PCR generated by BioNumerics using UPGMA. The genetic similarity between the isolates was 90% (dashed line).

Of all nine *bla*_OXA-48_-carrying *K. pneumoniae* isolates, two groups (cluster A and B) and a singleton were observed to have a 90% similarity of ERIC-PCR patterns (Figure 1). KP203, KP197, and KP166 isolates belonged to cluster A and exhibited a wide range of carbapenem susceptibility (imipenem MIC of 0.5, 8, and 32 mg/L). Five isolates, including KP221, KP260, KP1184, KP262, and KP241, belonged to cluster B with a broad range of imipenem MIC (0.5–128 mg/L) as well. KP162 isolate was resistant to all tested carbapenems and hence, was grouped as a unique singleton (Table 1). These results indicated that *bla*_OXA-48_-carrying *K. pneumoniae* outbreaks in our hospital have independently diverged from two major groups whose isolates possessed a variety of carbapenem susceptibility patterns from susceptible to resistant phenotypes.

### 2.3. OXA-48 Expression Level among Different Carbapenem-Resistant K. pneumoniae

**Figure 2:**
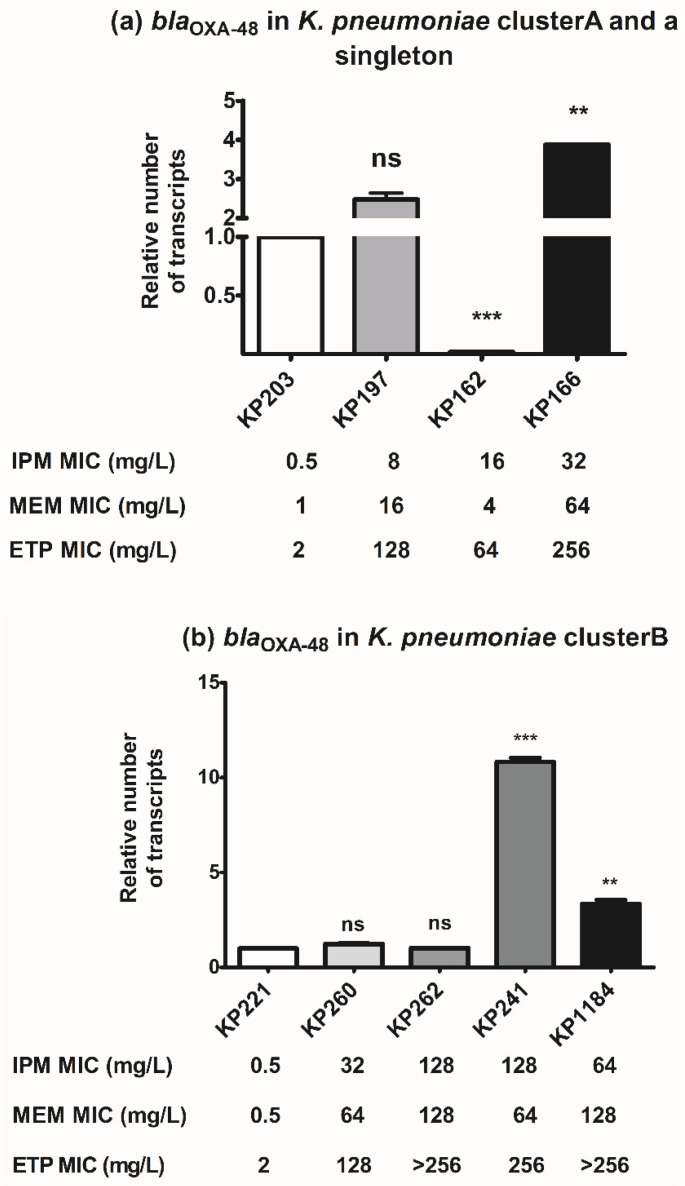
Relative *bla*_OXA-48_ expression levels of *K. pneumoniae*. RT-qPCR assay of *bla*_OXA-48_ expression was performed in *K. pneumoniae* cluster A (**a**), a singleton isolate KP162 (**a**), and cluster B (**b**). The relative number of *bla*_OXA-48_ transcripts of *K. pneumoniae* isolates was normalized to 16S rRNA expression and calculated using the 2^−^^ΔΔct^ method compared to the expression of imipenem-susceptible *K. pneumoniae* isolates KP203 and KP221. *p*-values were calculated using unpaired *t*-test (**, *p*-value < 0.01; ***, *p*-value < 0.001 and ns, non-significant).

All *bla*_OXA-48_-carrying *K. pneumoniae* isolates had comparatively different levels of carbapenem susceptibility. To investigate the correlation of *bla*_OXA-48_ with carbapenem resistance, *bla*_OXA-48_ expression among different clonal clusters was evaluated. In cluster A and a singleton (KP162), the relative expression of *bla*_OXA-48_ in each isolate was compared to that of imipenem-susceptible isolate, KP203. Overexpression of *bla*_OXA-48_ was observed in KP166 and KP197 isolates which exhibited imipenem MIC of 32 and 8 mg/L, respectively (Figure 2a), indicating that *bla*_OXA-48_ expression was probably related to carbapenem resistance level in *K. pneumoniae* cluster A. Of note, *bla*_OXA-48_ expression of KP162 isolate belonging to a singleton was significantly downregulated compared to that of KP203, indicating that other mechanisms played a role in carbapenem resistance of KP162 (Figure 2a). Among isolates belonging to cluster B, overexpression of *bla*_OXA-48_ was significantly observed in KP241 and KP1184 isolates whose imipenem MICs were 128 and 64 mg/L, respectively (Figure 2b). No significant expression of *bla*_OXA-48_ was observed in carbapenem-resistant KP260 and KP262 isolates compared to that of imipenem-susceptible KP221 isolate (Figure 2b). Therefore, among cluster B, expression levels were slightly related to carbapenem susceptibility in a few isolates. In conclusion, *bla*_OXA-48_ expression correlated to carbapenem resistance in some strains likely in a strain-specific manner, indicating the involvement of other mechanisms.

### 2.4. Impact of bla_OXA-48_-Carrying Plasmid in Carbapenem Susceptibility

Transformation of *bla*_OXA-48_-carrying plasmids to *E. coli* DH5α was used to investigate the impact of *bla*_OXA-48_-carrying plasmids on carbapenem susceptibility. The presence of *bla*_OXA-48_-carrying plasmid evidently increased imipenem MIC from 32-fold to 512-fold, whereas the MICs of meropenem and ertapenem appeared to raise at least 1024-fold in *E. coli* transformants (Table 2). Although KP162 isolate had a low level of *bla*_OXA-48_ expression, its plasmid showed remarkably increasing carbapenem MICs in the transformant (Figure 2a and Table 2). This was similar to imipenem-susceptible KP203 isolate that its transformant was resistant to all tested carbapenems (Table 2). Among cluster A, the transformants of KP166 and KP197 with high carbapenem resistance and *bla*_OXA-48_ expression had the same level of carbapenem resistance as that of imipenem-susceptible isolate KP203 (Table 2 and Figure 2a). Although isolates that belonged to cluster B (including KP221, KP260, and KP262) had near-identical expression level of *bla*_OXA-48_, they differed in carbapenem susceptibility (Figure 2b). Moreover, their transformants showed almost equal carbapenem MICs (Table 2). On the other hand, KP241 and KP1184 overexpressed *bla*_OXA-48_, but its transformants had the lowest carbapenem MICs (Figure 2b and Table 2). The results of *bla*_OXA-48_ expression and transformation indicate that apart from *bla*_OXA-48_, there may be other mechanisms involved in carbapenem resistance. Additionally, marked changes in the MICs of ceftriaxone, fosfomycin, and amikacin were also observed in all transformants (Table 2).

### 2.5. Loss of OmpK35 and/or OmpK36 among bla_OXA-48_-Producing K. pneumoniae

**Figure 3:**
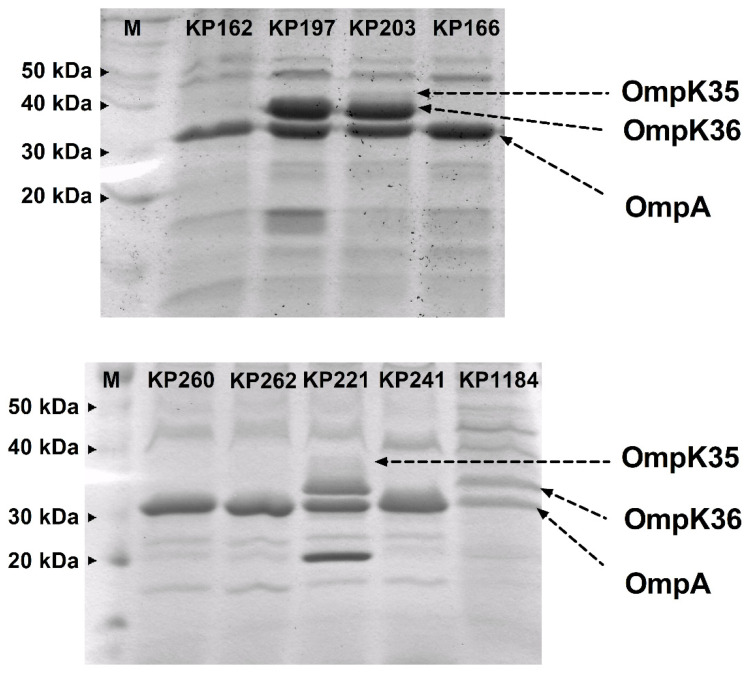
OMP profiles of nine OXA-48-producing *K. pneumoniae* isolates. OMP profiles were performed by SDS-PAGE. The arrow lines indicate OmpK35, OmpK36, and OmpA, respectively.

Since the results indicated the involvement of other resistance mechanisms, the loss of OMPs was investigated in all *bla*_OXA-48_-carrying isolates. The OMP profiles of all isolates are shown in Figure 3. Most isolates (KP162, KP166, KP260, KP262, and KP241), which lacked OmpK35 and OmpK36, were both low-level and high-level expressions of *bla*_OXA-48_. KP197 and KP1184 isolates showed slight *bla*_OXA-48_ overexpression with the loss of OmpK35 that had imipenem MICs of 8 and 64 mg/L, respectively (Figure 2b and Figure 3). Among isolates belonging to cluster A, KP166 that exhibited the highest carbapenem MIC (32–256 mg/L) and *bla*_OXA-48_ expression (Figure 2a), and was deficient in both OmpK35 and OmpK36 (Figure 3). A singleton, KP162, showed the lowest *bla*_OXA-48_ expression (Figure 2a) lacked both OmpK35 and OmpK36 (Figure 3). The presence of both OmpK35 and OmpK36 was only observed in imipenem- and meropenem-susceptible KP203 and KP221 belonging to clusters A and B, respectively. These results demonstrate that *bla*_OXA-48_ expression together with loss of OMPs, particularly OmpK36, have an affluential role on carbapenem susceptibility among *bla*_OXA-48_-carrying *K. pneumoniae* clinical isolates.

### 2.6. Ompk35 and Ompk36 Expression and Killing Effect of Imipenem against bla_OXA-48_-Carrying K. pneumoniae

**Figure 4:**
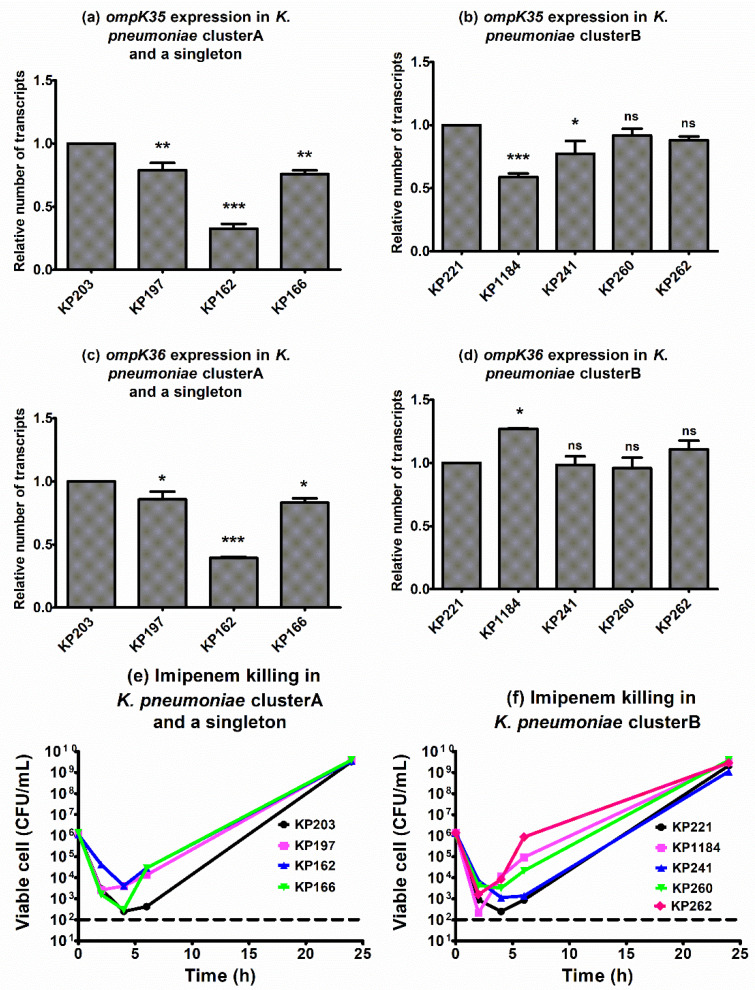
Relative *ompK35* and *ompK36* expression levels in *K. pneumoniae*. RT-qPCR assay of *ompK35* and *ompK36* expression was performed in *K. pneumoniae* cluster A (**a**,**c**), a singleton isolate KP162 (**a**,**c**), cluster B (**b**,**d**). The relative number of *ompK35* and ompK36 transcripts of *K. pneumoniae* isolates was normalized to 16S rRNA expression and calculated using the 2^−^^ΔΔct^ method compared to the expression of imipenem-susceptible *K. pneumoniae* isolates KP203 and KP221. *p*-values were calculated using unpaired *t*-test (*, *p*-value < 0.05; **, *p*-value < 0.01; ***, *p*-value < 0.001 and ns, non-significant). Killing effect of imipenem against *K. pneumoniae* cluster A and a singleton (**e**), and cluster B (**f**). Mean of bacterial viable cells at the treatment with 1 × MIC of imipenem was plotted at 0, 2, 4, 6, and 24 h of incubation. All experiments were performed in triplicate and the detection limit of the viable cells was 10^2^ CFU/mL (dashed lines).

Apart from Omp profile detection by SDS-PAGE, the expression levels of *ompK* genes and killing effects of imipenem were determined to correlate with the resistance mechanism. Among isolates in cluster A and a singleton, isolate KP162 showed significantly reduced expressions of *ompK35* and *ompK36* genes (Figure 4a,c). The most downregulation was of *ompK35* was KP1184 (Figure 4b), but ompK36 showed a similar expression level in cluster B (Figure 4d).

Time–killing curves of all isolates in the presence of 1 × MIC of imipenem exhibited the same patterns, which drastically killed within 2–4 h, followed by regrowth (Figure 4e,f). The carbapenem-resistant isolate KP162, which had the lowest expression of *bla*_OXA-48_ (Figure 2a), displayed the least of both *ompK35* and *ompK36* downregulations (Figure 4a,c), indicating the involvement of OmpK at least in part of carbapenem resistance.

### 2.7. Azithromycin and Fosfomycin Resistance and The Resistance Genes in bla_OXA-48_-Carrying K. pneumoniae

The range of azithromycin MIC of all nine *bla*_OXA-48_-carrying *K. pneumoniae* was 8–1024 mg/L. Most of the *K. pneumoniae* isolates were resistant to azithromycin (Table 1). However, azithromycin was still effective against isolates KP203 and KP1184 with MICs of 8 and 16 mg/L, respectively (Table 1). KP260 and KP262 were highly resistant with MIC of 1024 mg/L. The presence of erythromycin resistance methylase genes was determined by using PCR. All isolates carried *ermC* gene (Table 3). Moreover, high-level azithromycin-resistant isolates (KP260 and KP262) carried not only *ermC* but also *ermB*, and these isolates belonged to cluster B (Figure 1 and Table 3).

In addition, the presence of fosfomycin-modifying enzyme genes was detected in all *K. pneumoniae*, of which five and four isolates were susceptible and resistant to fosfomycin, respectively. The most common gene was *fosA5*, found in all isolates (Table 3). The coexistence of *fosA3* with *fosA5* was exhibited in five isolates (KP197, KP166, KP262, KP1184, and KP241).

### 2.8. Synergistic Activity of Azithromycin with Other Antibiotics against bla_OXA-48_-Carrying K. pneumoniae

The in vitro activities of azithromycin in combination with either imipenem, colistin, or fosfomycin against *bla*_OXA-48_-carrying *K. pneumoniae* were performed by using the checkerboard assay. Despite azithromycin resistance, the synergism was revealed in combination with colistin (88.89%), fosfomycin (77.78%), and imipenem (11.11%), respectively (Table 3). Azithromycin with colistin, the most effective combination, exhibited synergism in all isolates, except in *ermC*- and *ermB*-co-carrying KP262. Azithromycin and fosfomycin combination showed a synergistic effect in all isolates belonging to cluster A and a singleton that carried *ermC*. Remarkably, synergism of this combination was also observed against isolates in cluster B that carried *ermC* without *ermB*. The combination of azithromycin with imipenem had the least activity that was synergistic against only isolate KP1184 (Table 3). No antagonism was observed in our study.

### 2.9. Time–Kill Curves of Azithromycin Combination with Colistin against K. pneumoniae

**Figure 5:**
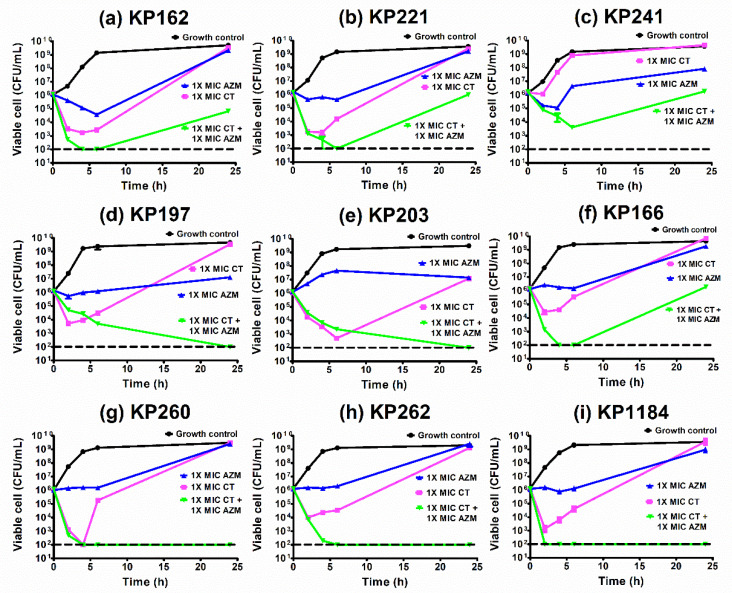
Time–kill curves of azithromycin and colistin combination against OXA-48-producing *K. pneumoniae* isolates. Synergism of azithromycin and colistin was confirmed by time–killing assays against KP162 (**a**), KP221 (**b**), KP241 (**c**), KP197 (**d**), KP203 (**e**), KP166 (**f**), KP260 (**g**), KP262 (**h**), and KP1184 (**i**). Mean of bacterial viable cells at each condition: growth control or without antibiotic (circles), colistin = 1 × MIC (squares), azithromycin = 1 × MIC (triangles), and colistin = 1 × MIC combination with azithromycin = 1 × MIC (upside down triangles) was plotted at 0, 2, 4, 6, and 24 h of incubation. All experiments were performed in triplicate and the detection limit of the viable cells was 102 CFU/mL (dashed lines).

According to checkerboard results, the most effective combination was azithromycin plus colistin. We, therefore, investigated the activity of this combination by time–killing assay. The time–killing curves of all isolates are shown in Figure 5. Although all isolates were susceptible to colistin, the regrowth was usually observed at 2–6 h after exposure to colistin alone (Figure 5a–i). This was similar to the presence of azithromycin alone in which the concentration of 1 × MIC could not eliminate the growth of both azithromycin-susceptible and azithromycin-resistant isolates. These results indicated that single use of either colistin or azithromycin may be inadequate for *bla*_OXA-48_-carrying *K. pneumoniae*. 

In combination, the synergism was observed in eight *bla*_OXA-48_-carrying *K. pneumoniae* isolates (KP162, KP221, KP197, KP203, KP166, KP260, KP262, and KP1184) (Figure 5). Notably, KP241 that showed synergy by checkerboard assay had no synergy result by time–killing assay and vice versa for KP262 isolate (Table 3, Figure 5c,h), indicating non-accordance of checkerboard and time–killing assay.

## 3. Discussion

Currently, carbapenem-resistant *K. pneumoniae*, an urgent threat, has spread worldwide, including Thailand [5,18,19,20]. The predominance of carbapenem-resistant *K. pneumoniae* in Thailand and any other country in Asia is NDM producers or NDM with OXA-48 co-producers, but OXA-48 alone producers are also reported [9,19]. OXA-48 producers exhibit a wide range of carbapenem susceptibility. The phenotype of *bla*_OXA-48_-carrying *K. pneumoniae* in our study is slightly in accordance with that of isolates from Taiwan that most isolates were resistant to carbapenems and all isolates were resistant to ertapenem [21]. The silence of *bla*_OXA-48_ was revealed in imipenem- and/or meropenem-susceptible *K. pneumoniae* isolates, including two isolates (KP203 and KP221) in our study [20]. Imipenem alone had inadequate activity in vivo against imipenem-susceptible isolates carrying *bla*_OXA-48_ and led to treatment failure [22,23]. These results indicate an inappropriate treatment for imipenem- and/or meropenem-susceptible *K. pneumoniae* carrying *bla*_OXA-48_ by carbapenems. In contrast, imipenem or meropenem monotherapy has been reported to be effective against OXA-48-producers [24]. Not only carbapenems but also other antibiotics have limited activities to *bla*_OXA-48_-carrying *K. pneumoniae* that are MDR strains. Fortunately, none of our isolates were resistant to the last line antibiotic, colistin. Colistin resistance became widespread among carbapenem-resistant *K. pneumoniae* [25]. According to the clonality in our study, this data indicates the clonal spread (ERIC-PCR cluster A and B) of *bla*_OXA-48_-carrying *K. pneumoniae* in our hospital. OXA-48 strongly hydrolyzes ertapenem rather than hydrolyzing imipenem and meropenem [26,27]. It is in accordance with our results that all *bla*_OXA-48_-carrying *K. pneumoniae* were resistant to ertapenem. However, phenotypes of imipenem and meropenem susceptibility were diverse among our isolates, indicating the involvement of other resistance determinants apart from *bla*_OXA-48_ expression level. *E. coli* transformants carrying *bla*_OXA-48_-plasmids isolated from all *K. pneumoniae* isolates displayed a rising of carbapenem MICs. This experiment indicated that different plasmids displayed different levels of carbapenem resistance. Nevertheless, colistin with azithromycin remained effective against almost all of these isolates. In this study, we used *E. coli* DH5α as a recipient cell due to lacking *K. pneumoniae* competent cells. Making in-house competent cells is arduous, and *K. pneumoniae* reference strains are unavailable in our facility. Although they are different species, the spread of these plasmids generally occurs between *K. pneumoniae* and *E. coli* [28].

Porins, OMPs acting as pores that the specific substrates can diffuse into intracellular compartment, operating as carbapenem entries in *K. pneumoniae* are OmpK35 and OmpK36 [10]. Loss of OmpK35 results in increase imipenem and meropenem MICs in *K. pneumoniae* producing ESBLs [2,29]. The deletion of *ompK35* results in 2-fold and 4-fold increase of imipenem and ertapenem MICs, respectively, but no change of these MICs is observed in *ompK36* deletion strain indicating that OmpK35 plays a superior role to OmpK36 in carbapenem susceptibility [30]. This is similar to our results which showed that isolates lacking OmpK35 or lacking both OmpK35 and OmpK36 had no difference in carbapenem resistance levels (Table 3). However, loss of OmpK36 together with KPC production confers resistance to carbapenem in *K. pneumoniae* [10,31]. The mutations of OmpK36, particularly, insertion of Gly115-Asp116 into loop 3 with KPC or OXA-48 production resulted in the elevation of carbapenem MICs [32]. It is in agreement with our results which showed that higher resistance to carbapenems was found in isolates with loss of OmpK35 and OmpK36 compared to isolates with intact OMPs. Thereby, OXA-48 production with lacking OMPs affects the magnitude of carbapenem resistance in *bla*_OXA-48_-carrying *K. pneumoniae* isolates. Isolate KP162, which was resistant to carbapenem with the lowermost expression of *bla*_OXA-48_ (Figure 2a), also showed the most downregulation of *ompK35* and *ompK36* (Figure 4a,c). Although all isolates were sharply killed in early exposure to imipenem (2–4 h), the regrowth usually occurred after 6 h (Figure 4e,f), indicating that imipenem alone is inadequate for treatment of *bla*_OXA-48_-producing *K. pneumoniae*. Notably, the results of the transformation experiment demonstrated that the *E. coli* transformants were resistant to not only carbapenems but also other antibiotics, indicating multiple resistance gene-carrying plasmids. This data supported the evidence that *bla*_OXA-48_-carrying *K. pneumoniae* frequently were multidrug-resistant organisms. 

Due to multidrug resistance in *bla*_OXA-48_-carrying *K. pneumoniae*, the treatment options were limited. Antibiotic combination therapy is inevitably used during a shortage of novel effective antibiotics. The main aim of this study was to forage and obtain the combinations of antibiotics that may have a potential effect against *bla*_OXA-48_-carrying *K. pneumoniae*. Azithromycin, a macrolide antibiotic that inhibits translocation and transpeptidation of protein synthesis by binding to 50S ribosomal subunit at 23S rRNA [15], was chosen to be combined with other commonly used antibiotics (imipenem, colistin, and fosfomycin). The majority of isolates in our study were resistant to azithromycin, indicating an ineffective effect of azithromycin in single use against *bla*_OXA-48_-carrying *K. pneumoniae*. All isolates carried *ermC* alone or with *ermB*. These azithromycin resistance genes are generally found in Gram-positive cocci and spread to Gram-negative bacilli by plasmid-mediated horizontal gene transfer [33,34]. 

Remarkably, in our study, azithromycin with colistin was the most potent combination observed by checkerboard assay, and its synergism was confirmed by the time–killing assay against *bla*_OXA-48_-carrying *K. pneumoniae*. Furthermore, we performed the in vitro activity of this combination against additionally 26 carbapenem-resistant *K. pneumoniae* clinical isolates producing various patterns of carbapenemase. The synergism was observed in 15 isolates (57.69%) (Supplementary Materials Table S1). Interestingly, only half of NDM producers (6 of 10 isolates) and NDM with OXA-48 producers (6 of 12 isolates) showed synergism of the antibiotic combination (Table S1). The in vitro synergism of this combination has been reported against MDR *P. aeruginosa*, MDR-*Acinetobacter baumannii*, colistin-resistant *E. coli*, and MDR-*K. pneumoniae* [16,35]. The synergistic mechanism of the combination is revealed in MDR-*A. baumannii* that colistin, like antimicrobial peptide, disrupts the bacterial cell membrane and enhances azithromycin uptake, resulting in bacterial death [16]. The limitation of our study was that the synergistic mechanism of azithromycin and colistin was not performed. Moreover, the purpose of using azithromycin, especially in cystic fibrosis caused by *P. aeruginosa*, is to reduce inflammation response and to inhibit biofilm formation, not to kill the bacteria [36]. Recently, azithromycin has been used as an immunomodulator to modulate immune response to respiratory tract infection [37].

Additionally, synergism was remarkably observed in the combination of azithromycin and fosfomycin. Our previous study demonstrates that fosfomycin resistance genes (*fosA5* and *fosA3*) silence in carbapenem-resistant *K. pneumoniae* (including *bla*_OXA-48_-carrying isolates) probably leading to insufficient activity [9]. Therefore, the activity of azithromycin with fosfomycin was not determined by time–killing assay. Moreover, amikacin seemed effective against OXA-48-producing strains. Unfortunately, amikacin-heteroresistant subpopulations have been reported among amikacin-susceptible populations, plausibly leading to treatment failure [38]. Therefore, amikacin was not included in the combination testing in this study.

## 4. Materials and Methods

### 4.1. Bacterial Strains

Thirty-five OXA-48-carrying *K. pneumoniae* isolated from nonduplicate patients were collected from the routine laboratory’s stocks at the King Chulalongkorn Memorial Hospital, Bangkok, Thailand, from 2017–2018. Our study was approved by the Institutional Review Board of Faculty of Medicine, Chulalongkorn University (IRB 221/62). Neither human nor animal was involved in this study. The need for consent was waived by the ethics committee. 

### 4.2. Antibiotic Susceptibility Testing

All antibiotic susceptibilities were performed according to the European Committee on Antimicrobial Susceptibility Testing (EUCAST) guidelines [39]. The minimum inhibitory concentrations (MICs) of imipenem (Apollo Scientific, Manchester, UK), meropenem (Sigma-Aldrich, Steinheim, Germany), ertapenem (MSD, Kenilworth, NJ, USA), ceftriaxone (Sigma-Aldrich, Steinheim, Germany), and amikacin (Sigma-Aldrich, Germany) were determined by using broth microdilution method with cation-adjust Mueller–Hinton broth (CAMHB) (Becton Dickenson BBL, Sparks, MD, USA). Fosfomycin (Wako Pure Chemical Industries, Osaka, Japan) susceptibility was performed by agar dilution method using Mueller–Hinton agar supplemented with 25 mg/L of glucose-6-phosphate (G6P) (Sigma-Aldrich, Steinheim, Germany). Azithromycin (Sigma-Aldrich, Steinheim, Germany) susceptibility test was determined by broth microdilution method. *E. coli* ATCC 25922 and *P. aeruginosa* ATCC 27853 were used as reference control strains for susceptibility testing. The antibiotic susceptibility was interpreted according to EUCAST guidelines (Supplementary Materials Table S2). 

### 4.3. Detection of Antibiotic Resistance Genes

Multiplex PCR was used for the detection of carbapenemase genes including *bla*_NDM-like_, *bla*_OXA-48-like_, and *bla*_KPC-like_ as described by Poirel et al. [40]. The presence of metallo-carbapenemase genes including *bla*_IMP-like_ and *bla*_VIM-like_ was performed by using multiplex PCR as described by Ellington et al. [41]. Fosfomycin-modifying enzyme genes including *fosA5* and *fosA3* were detected as in a previous study [9]. Erythromycin resistance methylase genes including *ermA*, *ermB*, *ermC*, and *ermF* were detected by PCR [42,43,44]. The primers used in this study are listed in Table S3.

### 4.4. Clonal Study

The genetic relatedness of nine OXA-48-producing *K. pneumoniae* isolates was characterized by the Enterobacterial Repetitive Intergenic Consensus (ERIC) PCR (ERIC-PCR) [45]. The dendrogram of ERIC-PCR was generated by BioNumerics software, version 8.0, using the UPGMA. Clonal relatedness was defined as >90% similarity. 

### 4.5. Expression Level of bla_OXA-48_, ompK35 and ompK36

The expression level of *bla*_OXA-48_, *ompK35*, and *ompK36* mRNA was studied by RT-qPCR. Total RNA of nine *bla*_OXA-48_-carrying *K. pneumoniae* was extracted by using TRIzol^®^ Reagent (Invitrogen, Carlsbad, CA, USA). cDNA was synthesized by using RevertAid First Strand cDNA Synthesis Kit (Thermo Scientific, Vilnius, Lithuania). The number of *bla*_OXA-48_ transcripts was determined by using the Luna^®^ Universal qPCR master and the QuantStudio5 (Applied Biosystems, Foster City, CA USA). The relative number of *bla*_OXA-48_ transcripts was normalized with 16S rRNA and determined by using the 2^−^^ΔΔct^ method. This experiment was performed in triplicate. 

### 4.6. Transformation of the bla_OXA-48_-Carrying Plasmids into E. coli DH5α

To investigate the role of *bla*_OXA-48_-carrying plasmids isolated from *K. pneumoniae* on carbapenem susceptibility, the plasmid was extracted by using HiYield Plasmid Mini Kit (RBC, New Taipei City, Taiwan) and transformed into *E. coli* DH5α by using the heat shock method. *E. coli* DH5α transformants were selected on MHA supplemented with imipenem. The transformants were confirmed the presence of *bla*_OXA-48_ plasmid by PCR and tested for antimicrobial susceptibility by broth microdilution method. 

### 4.7. Outer Membrane Protein (OMP) Study

To study OMP profiles of *bla*_OXA-48_-carrying *K. pneumoniae*, OMPs were extracted by ultracentrifugation with *N*-Lauroylsarcosine (Merck Millipore, Kenilworth, NJ, USA) extraction as previously described [46]. Briefly, log-phase growth of *K. pneumoniae* in Nutrient Broth (Becton Dickenson Difco, Sparks, MD, USA) was broken by sonication (Sonics and Materials, Inc., Newtown, CT, USA). OMPs were extracted with *N*-Lauroylsarcosine solution and collected by ultracentrifugation. OMP profile was determined by SDS-PAGE. 

### 4.8. Checkerboard Assay

The activity of azithromycin in combination with other antimicrobial agents including imipenem, colistin, and fosfomycin, was determined by checkerboard assay in 96-well culture plates. Each well of each column contained CAMHB supplemented with two-fold serial dilution of azithromycin, whereas the serial dilution of either imipenem, colistin, or fosfomycin was added in each well of each row. In the case of fosfomycin, 25 mg/L of G6P was also added. *K. pneumoniae* was inoculated into the checkerboard plates, and the plates were incubated for 18–24 h at 37 °C. The fractional inhibitory concentration index (FICI) was calculated using the following Equation:

$$\mathrm{FICI}=\frac{\mathrm{MIC}\;\mathrm{of}\;\mathrm{drug}\;A\;\mathrm{in}\;\mathrm{combination}}{\mathrm{MIC}\;\mathrm{of}\;\mathrm{drug}\;A\;\mathrm{alone}}+\frac{\mathrm{MIC}\;\mathrm{of}\;\mathrm{drug}\;B\;\mathrm{in}\;\mathrm{combination}}{\mathrm{MIC}\;\mathrm{of}\;\mathrm{drug}\;B\;\mathrm{alone}}$$

The interpretation was defined as following, synergism (FICI ≤ 0.5), no interaction (FICI > 0.5–4), and antagonism (FICI > 4).

### 4.9. Time–Killing Assay

The killing effect of imipenem alone was performed by the time–killing assay. Briefly, viable cells of *K. pneumoniae* exposure to 1 × MIC of imipenem were determined after incubation time for 0, 2, 4, 6, and 24 h at 37 °C with shaking were counted on MHA plates. The synergism of azithromycin in combination with colistin was performed by the time–killing assay. Briefly, viable cells of *K. pneumoniae* in different conditions including growth control (no antibiotic), 1 × MIC of azithromycin, 1 × MIC of colistin, and 1 × MIC of azithromycin plus 1 × MIC of colistin after incubation for 0, 2, 4, 6, and 24 h at 37 °C with shaking were counted on MHA plates. This experiment was performed in triplicate. The synergism was defined as the reduction of the viable cell at least 2log10-fold compared to the most effective single antibiotic at 24 h of incubation. The bactericidal activity was defined as the reduction of the viable cell at least 3log10-fold compared to the initial viable cell after 24 h of incubation. 

### 4.10. Statistical Analysis

The statistical analysis was performed by with GraphPad Prism 5 (unpaired *t*-test) (GraphPad Software, San Diego, CA, USA).

## 5. Conclusions

Among two major clonal spreading events, *bla*_OXA-48_-carrying *K. pneumoniae* was responsible for a wide range of carbapenem MICs, especially imipenem MIC. Imipenem-susceptible isolates had intact OmpK35 and OmpK36. OXA-48 production with lacking OMPs resulted in high resistance to carbapenems. The most effective combination was azithromycin with colistin. Although azithromycin is not currently used to treat *K. pneumoniae*, its combination with colistin may provide a potential activity for *bla*_OXA-48_-carrying *K. pneumoniae*. Further in vivo study is needed to assess the application of this antibiotic combination. Our results highlighted azithromycin for *bla*_OXA-48_-carrying *K. pneumoniae* treatment at least in part of novel combination therapy and knowledge for novel antibiotic development. 

## Figures and Tables

**Table 1:** Antibiotic susceptibility of nine OXA-48-producing *K. pneumoniae* isolates.

Isolate	MIC (Mg/L) (Interpretation)								
	IMP	MEM	ETP	AMK	CIP	CRO	CT	FOF	AZM
KP162	16 (R)	4 (I)	64 (R)	4 (S)	128 (R)	128 (R)	0.5 (S)	128 (R)	64 (R)
KP166	32 (R)	64 (R)	256 (R)	4 (S)	1 (R)	>256 (R)	0.25 (S)	256 (R)	32 (R)
KP197	8 (R)	16 (R)	128 (R)	16 (R)	64 (R)	>256 (R)	0.5 (S)	32 (S)	32 (R)
KP203	0.5 (S)	1 (S)	2 (R)	4 (S)	128 (R)	128 (R)	0.5 (S)	16 (S)	8 (S)
KP221	0.5 (S)	0.5 (S)	2 (R)	1 (S)	128 (R)	128 (R)	0.5 (S)	8 (S)	128 (R)
KP241	128 (R)	64 (R)	256 (R)	2 (S)	64 (R)	>256 (R)	0.25 (S)	128 (R)	128 (R)
KP260	32 (R)	64 (R)	128 (R)	4 (S)	>256 (R)	>256 (R)	0.5 (S)	256 (R)	1024 (R)
KP262	128 (R)	128 (R)	>256 (R)	8 (S)	>256 (R)	>256 (R)	0.25 (S)	1024 (R)	1024 (R)
KP1184	64 (R)	128 (R)	>256 (R)	64 (R)	64 (R)	>256 (R)	1 (S)	16 (S)	16 (S)

IPM: Imipenem; MEM: Meropenem; ETP: Ertapenem; AMK: Amikacin; CIP: Ciprofloxacin; CRO: Ceftriaxone; CT: Colistin; FOF: Fosfomycin; AZM: Azithromycin; S: Susceptible; I: Intermediate resistant; R: Resistant.

**Table 2:** Antibiotic susceptibility of nine OXA-48-producing *K. pneumoniae* isolates and *E. coli* DH5α transformants.

Isolate	MIC (mg/L)						
	Carbapenemase Gene	IPM	MEM	ETP	CRO	FOF	AMK
*E. coli* DH5α	None	0.25	0.015	0.015	0.015	0.5	0.5
KP162	*bla*_OXA-48_	16	4	64	128	128	4
KP162_T	*bla*_OXA-48_	128	64	>256	>256	>256	8
KP197	*bla*_OXA-48_	8	16	128	>256	32	16
KP197_T	*bla*_OXA-48_	8	16	16	32	2	1
KP203	*bla*_OXA-48_	0.5	1	2	128	16	4
KP203_T	*bla*_OXA-48_	64	32	256	>256	>256	2
KP166	*bla*_OXA-48_	32	64	256	>256	256	4
KP166_T	*bla*_OXA-48_	64	32	256	>256	256	2
KP260	*bla*_OXA-48_	32	64	12	>256	256	4
KP260_T	*bla*_OXA-48_	16	16	16	16	4	1
KP262	*bla*_OXA-48_	128	128	>256	>256	256	8
KP262_T	*bla*_OXA-48_	64	32	256	>256	128	2
KP1184	*bla*_OXA-48_	64	128	>256	>256	>256	64
KP1184_T	*bla*_OXA-48_	8	16	16	32	4	1
KP221	*bla*_OXA-48_	0.5	0.5	2	128	8	1
KP221_T	*bla*_OXA-48_	16	16	16	16	4	1
KP241	*bla*_OXA-48_	128	64	256	>256	128	2
KP241_T	*bla*_OXA-48_	8	16	16	16	4	1

T: *E. coli* DH5α Transformant; IPM: Imipenem; MEM: Meropenem; ETP: Ertapenem; CRO: Ceftriaxone; FOF: Fosfomycin; AMK: Amikacin.

**Table 3:** The synergistic activity of azithromycin in combination with other antibiotics against OXA-48-producing *K. pneumoniae* isolates.

Isolate	OMP		MIC (Mg/L) (Interpretation)				Antibiotic Resistance Gene				FICI (Interpretation)		
	Ompk35	Ompk36	IPM	CT	FOF	AZM	*fosa3*	*fosa5*	*ermB*	*ermC*	AZM + IPM	AZM + CT	AZM + FOF
KP162	−	−	16 (R)	0.5 (S)	128 (R)	64 (R)	−	+	−	+	0.75 (N)	0.5 (Syn)	0.38 (Syn)
KP197	−	+	8 (R)	0.5 (S)	32 (S)	32 (R)	+	+	−	+	2 (N)	0.5 (Syn)	0.5 (Syn)
KP203	+	+	0.5 (S)	0.5 (S)	16 (S)	8 (S)	−	+	−	+	2 (N)	0.5 (Syn)	0.38 (Syn)
KP166	−	−	32 (R)	0.25 (S)	256 (R)	32 (R)	+	+	−	+	0.75 (N)	0.5 (Syn)	0.38 (Syn)
KP260	−	−	32 (R)	0.5 (S)	256 (R)	1024 (R)	−	+	+	+	2 (N)	0.38 (Syn)	0.75 (N)
KP262	−	−	128 (R)	0.25 (S)	256 (R)	1024 (R)	+	+	+	+	0.75 (N)	0.75 (N)	0.75 (N)
KP1184	−	+	64 (R)	1 (S)	512 (R)	16 (S)	+	+	−	+	0.5 (Syn)	0.38 (Syn)	0.38 (Syn)
KP221	+	+	0.5 (S)	0.5 (S)	8 (S)	128 (R)	−	+	−	+	0.75 (N)	0.38 (Syn)	0.38 (Syn)
KP241	−	−	128 (R)	0.25 (S)	128 (R)	128 (R)	+	+	−	+	0.75 (N)	0.5 (Syn)	0.38 (Syn)

FICI: Fractional Inhibitory Concentration Index; IPM: Imipenem; CT: Colistin; FOF: Fosfomycin; AZM: Azithromycin; −: Absence; +: Presence; R: Resistant; S: Susceptible; Syn: Synergism; N: No interaction.

## Data Availability

Data is contained in this manuscript or Supplementary Materials.

## Supplementary Materials

The following are available online at https://www.mdpi.com/article/10.3390/antibiotics10121551/s1, Table S1: The synergistic activity of azithromycin in combination with colistin against additionally 26 carbapenemase-producing *K. pneumoniae* isolates, Table S2: Interpretation of antibiotic susceptibility by MIC breakpoints, Table S3: Oligonucleotide sequences of primers used in this study. References [47,48,49] are cited in the supplementary materials.
