# Supplementary material for: In Vitro Synergism of Azithromycin Combination with Antibiotics against OXA-48-Producing Klebsiella pneumoniae Clinical Isolates
Source: Antibiotics (Basel). 2021 Dec 17;10(12):1551. doi: 10.3390/antibiotics10121551 (PMC8698995; doi:10.3390/antibiotics10121551)
Supplement: Supplementary file 1 [file antibiotics-10-01551-s001.zip › antibiotics-1505917-supplementary.pdf]

Supplementary Materials

Table S1. The synergistic activity of azithromycin in combination with colistin against additionally 26 carbapenemase-producing *K. pneumoniae* isolates.

Isolate	Carbapenemase	MIC (mg/L)				AZM + CT
		IPM	MEM	CT	AZM	FICI (Interpretation)
KP1	NDM	32	64	1	64	0.38 (Syn)
KP2	NDM OXA-48	64	128	1	64	0.31 (Syn)
KP3	NDM OXA-48	128	128	1	32	0.38 (Syn)
KP4	NDM OXA-48	64	128	32	32	0.14 (Syn)
KP5	NDM	256	256	64	32	0.13 (Syn)
KP6	NDM OXA-48	128	128	0.5	64	0.50 (Syn)
KP7	NDM OXA-48	>256	256	32	32	0.26 (Syn)
KP8	NDM	64	128	0.5	32	0.75 (N)
KP9	OXA-48	16	32	1	64	0.31 (Syn)
KP10	OXA-48	8	32	1	256	0.31 (Syn)
KP11	NDM	64	128	32	32	0.19 (Syn)
KP12	NDM	64	128	32	16	0.31 (Syn)
KP13	NDM	16	32	0.25	32	1.00 (N)
KP14	IMP	32	16	1	32	0.50 (Syn)
KP15	NDM	32	16	1	32	0.31 (Syn)
KP16	NDM OXA-48	32	128	0.5	128	0.50 (Syn)
KP17	NDM OXA-48	16	16	0.25	128	1.00 (N)
KP18	NDM OXA-48	4	8	0.25	256	1.00 (N)
KP19	NDM OXA-48	8	8	0.5	256	0.75 (N)
KP20	NDM	128	32	0.5	256	1.00 (N)
KP21	NDM	32	32	1	128	0.75 (N)
KP22	OXA-48 IMP	1	2	0.25	256	1.00 (N)
KP23	NDM OXA-48	64	128	0.25	64	0.75 (N)
KP24	NDM	16	16	0.5	64	0.50 (Syn)
KP25	NDM OXA-48	16	16	0.25	16	0.63 (N)
KP26	NDM OXA-48	64	128	0.5	64	0.56 (N)

IPM: Imipenem; MEM: Meropenem; CT: Colistin; AZM: Azithromycin, FICI: Fractional Inhibitory Concentration Index; Syn: Synergism; N: No interaction.

Table S2. Interpretation of antibiotic susceptibility by MIC breakpoints.

Antibiotic	MIC breakpoints (mg/L)		Note
	Susceptible (≤)	Resistant (>)	
Imipenem	2	4	
Meropenem	2	8	
Ertapenem	0.5	0.5	
Ciprofloxacin	0.25	0.5	
Colistin	2	2	
Ceftriaxone	1	2	
Fosfomycin	32	32	*E. coli* criteria
Azithromycin	16		*Salmonella* Typhi and *Shigella* species criteria
Amikacin	8	8	

Table S3. Oligonucleotide sequences of primers used in this study.

Gene	Primer sequences	PCR product size (bp)	Application	Reference
fosA	F- ATCTGTGGGTCTGCCTGTCGT	271	PCR	Lu et al. [47]
	R- ATGCCCGCATAGGGCTTCT			
fosA3	F- CGGAGCCTATCTCTCCTGTG	219	PCR	Singkham-in et al. [9]
	R- CCGTCAGGGTCGAGAAAATA			
fosA5	F- GCGCCGAGCGTGGCGTTT	283	PCR	Singkham-in et al. [9]
	R- GCCATCGGGATCGAGGAA			
fosB	F- AGGTGAGACCTCGGCCTATT	302	PCR	Singkham-in et al. [9]
	R- GAGGTTTAGCCTCTTTATAA-TAACTCA			
fosC2	F- GGGCATATCTGAGCTTGGAG	212	PCR	Singkham-in et al. [9]
	R- CAATTTATGGCCGTCAGGAT			
fosX	F- GTTGCGTTTAAGGCAGGAAG	425	PCR	Singkham-in et al. [9]
	R- GGCTCCATTTGTTGGACAGT			
ermA	F- ACGATATTCACGGTTTACCCACTTA	610	PCR	Khan et al. [44]
	R- AACCAGAAAAACCCTAAAGA-CACG			
ermB	F- GAAAAGGTACTCAACCAAATA	639	PCR	Sutcliffe et al. [42]
	R- AGTAACGGTACTTAAATTGTTTAC			
ermC	F- AGTACAGAGGTGTAATTTCG	520	PCR	Khan et al. [44]
	R- AATTCCTGCATGTTTTAAGG			
ermF	F- CGGGTCAGCACTTTACTATTG	468	PCR	Roberts et al. [43]
	R- GGACCTACCTACCTCATAGACAAG			
$bla_{OXA\text{-}48\text{-}like}$	F- GCGTGGTTAAGGATGAACAC	438	PCR / RT-qPCR	Poirel et al. [40]
	R- CATCAAGTTCAACCCAACCG			
ompK35	F- GTCGAAGCGGCAACCGATTATG	320	RT-qPCR	Hamzaoui et al. [48]
	R- GCTTCGGCTTTGTCGCCATT			
ompK36	F- GAGTTGCGTTGTAGGTCTGG	393	RT-qPCR	Hamzaoui et al. [48]
	R- GGCGACACCTACGGTTCTGACAA			
16S rRNA	F- GGAGGAAGGTGGGGATGACG	241	RT-qPCR	Hou et al. [49]
	R- ATGGTGTGACGGGCGGTGTG			
